# Right Gastroepiploic Artery as an Alternative for Arterial Reconstruction in Living Donor Liver Transplantation

**DOI:** 10.1155/2014/616251

**Published:** 2014-11-16

**Authors:** Klaus Steinbrück, Reinaldo Fernandes, Marcelo Enne, Rafael Vasconcelos, Giuliano Bento, Gustavo Stoduto, Thomas Auel, Lúcio Filgueiras Pacheco-Moreira

**Affiliations:** ^1^Hepatobiliary Surgery Unit, Serviço de Cirurgia Hepato-Biliar, Bonsucesso Federal Hospital-Health Ministry, Avenida Londres 616, Predio 3/2° Andar, 21041-030 Rio de Janeiro, RJ, Brazil; ^2^Transplantation Unit, São Francisco Hospital-Rio de Janeiro State Health Secretary, Rua Conde de Bonfim 1033, 20530-190 Rio de Janeiro, RJ, Brazil; ^3^General Surgery Department, Ipanema Federal Hospital-Health Ministry, Rua Antônio Parreiras 67/69, 22411-020 Rio de Janeiro, RJ, Brazil

## Abstract

*Background*. An adequate blood flow is directly related to graft survival in living donor liver transplantation. However, in some cases, unfavorable conditions prevent the use of the hepatic artery for arterial reconstruction. Herein, we report a case in which the recipient right gastroepiploic artery was used as an option for arterial reconstruction in adult-to-adult living donor liver transplantation. *Case Report*. A 62-year-old woman, with cirrhosis due to hepatitis B associated with hepatocellular carcinoma, was submitted to living donor liver transplantation. During surgery, thrombosis of the hepatic artery with intimal dissection until the celiac trunk was observed, which precluded its use in arterial reconstruction. We decided to use the right gastroepiploic artery for arterial revascularization of the liver graft. Despite the discrepancy in size between donor hepatic artery and recipient right gastroepiploic artery, anastomosis was performed successfully. *Conclusions*. The use of the right gastroepiploic artery as an alternative for arterial revascularization of the liver graft in living donor liver transplantation should always be considered when the hepatic artery of the recipient cannot be used. For performing this type of procedure, familiarity with microsurgical techniques by the surgical team is necessary.

## 1. Introduction

An adequate blood flow is directly related to graft survival and prevention of postoperative complications in living donor liver transplantation (LDLT). However, in some cases, unfavorable conditions prevent the use of the recipient hepatic artery for arterial reconstruction. In these cases, an alternative source for arterial inflow is necessary. Herein, we report a case in which the right gastroepiploic artery (RGEA) was used as an option for arterial reconstruction in adult-to-adult LDLT.

## 2. Case Report

**Figure 1:**
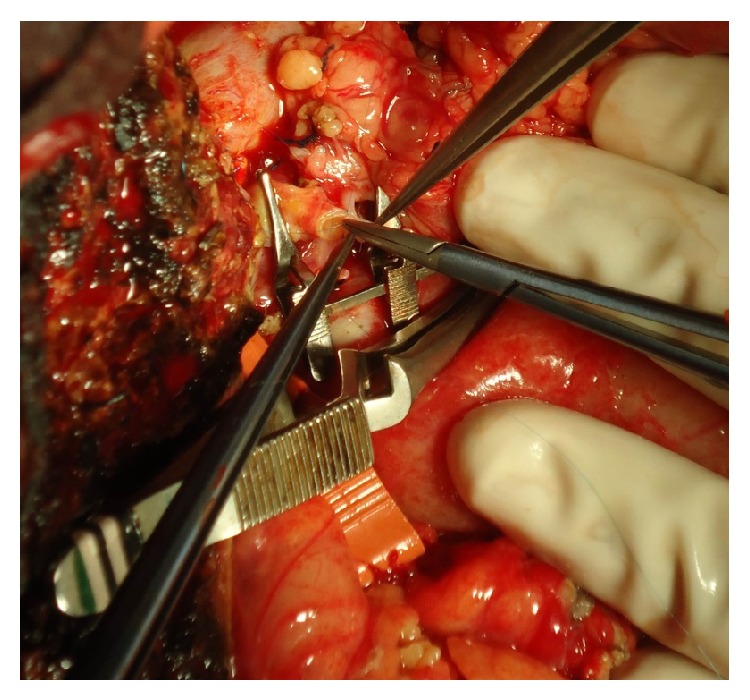
Performing anastomosis between donor right hepatic artery and recipient RGEA, using parallel bulldog clamp.

**Figure 2:**
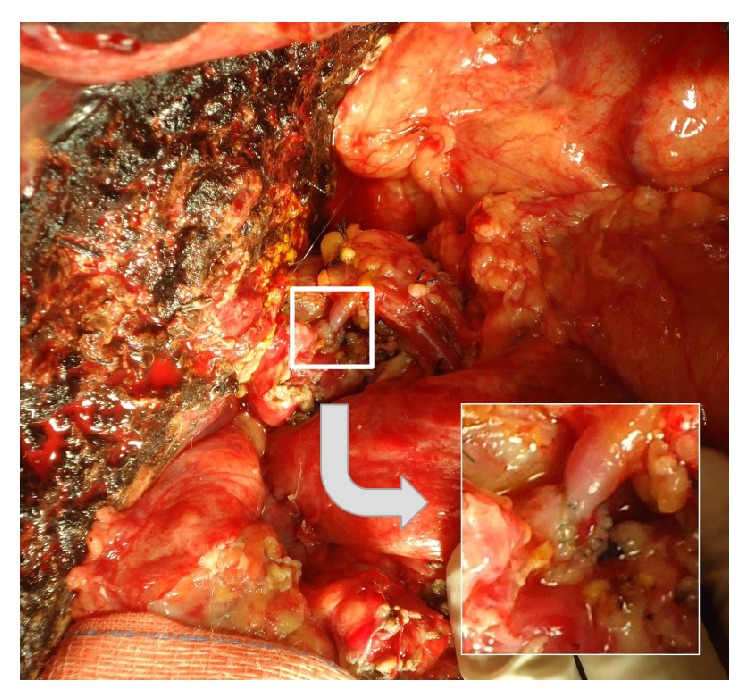
Final aspect, end-to-end anastomosis.

**Figure 3:**
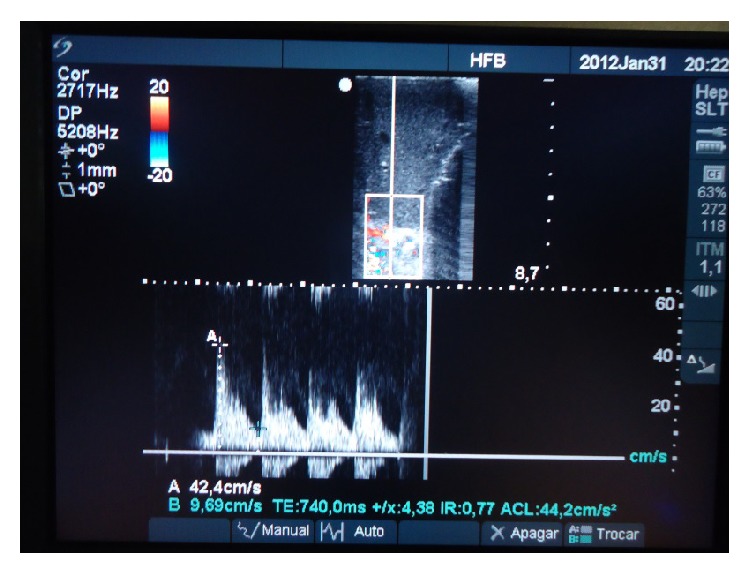
Intraoperative Doppler exam confirming intrahepatic arterial flow.

A 62-year-old woman, with cirrhosis due to hepatitis B associated with hepatocellular carcinoma, was submitted to adult-to-adult LDLT, using a right liver graft. Recipient, donor, and graft weight were 51 Kg, 72 Kg, and 774 g, respectively. Graft to recipient weight ratio was 1.52%. During recipient's hepatectomy, thrombosis of the hepatic artery with extensive subintimal dissection until celiac trunk was observed. The use of hepatic artery for graft revascularization was judged impossible. We decided to use the RGEA for arterial reconstruction. The artery was released from the gastric greater curvature, mobilized under the gastric pylorus, and approximated without tension to the liver graft. Despite the discrepancy in size between the donors right hepatic artery and the recipient RGEA, arterial anastomosis was performed successfully in an end-to-end fashion using separate 8–0 Prolene sutures. Magnification loupes (6x) were worn by surgeons. A parallel bulldog clamp was used to approximate the arteries during anastomosis (Figures 1 and 2). Intrahepatic blood flow was confirmed by intraoperative Doppler (Figure 3). During postoperative period, patency of arterial anastomosis was evaluated daily by Doppler exam. Patient was discharged on the 16th postoperative day and is in good general condition, 34 months after transplantation.

## 3. Discussion

The first description of RGEA as an alternative for liver graft revascularization in orthotopic liver transplantation was made by Radermecker et al. in 1993 considering a whole liver transplantation from deceased donor [1]. In 2000, Ikegami et al. [2] reported the use of this technique in adult-to-adult LDLT. In the same year, Itabashi et al. [3] performed the same method in a pediatric recipient with living donor.

With the development of liver transplantation, especially in transplant centers in Asia, other arteries have been described as alternative for arterial anastomosis in LDLT, such as splenic, left gastric, right gastric, middle colic, cystic, and gastroduodenal [4]. Still, RGEA remains the main option, when the hepatic artery cannot be used [5–7]. The preference for the RGEA can be explained by its easy and safe dissection, which causes no ischemia of the stomach. Furthermore, it is long enough to perform anastomosis without tension [7].

In the case presented here, as soon as we realized that the recipient right hepatic artery had severe thrombosis with wall dissection to the celiac trunk, we chose to use the RGEA for arterial reconstruction. The RGEA showed approximately 2 mm in diameter, slightly thinner than the recipient right hepatic artery. Despite the difference in size, arterial anastomosis was performed successfully using microsurgery techniques.

## 4. Conclusion

Liver transplantation teams ought to be prepared for unexpected situations during surgery. Right gastroepiploic artery should always be considered as an alternative for arterial reconstruction when the hepatic artery cannot be used. For performing this type of procedure, knowledge of microsurgical techniques by the surgical team is necessary.
